# Collectanea

**Published:** 1833-06

**Authors:** 


### COLLECTANEA.

### PATHOLOGY.

*Emphysematous Tumor of the Neck, accompanied by remarkable Phenomena.* By Dr. OLLIVIER, of Angers. [*Archives,* March 1833.]

M. R., of a sanguine and nervous temperament, and robust constitution, and generally in good health, repaired one morning to the house of a friend on business; he sat down quietly in an armchair; and, on throwing his head back to rest it against the chair, he suddenly felt a cracking sensation, deep-seated, on the right side of the neck, accompanied by slight pain. At the same moment a tumor formed in the part about the size of two fists, producing the greatest anxiety, sickness at the stomach, and a partial loss of consciousness. The friends thought the patient was labouring under apoplexy, and were anxious to have him bled; but Mr. R. very shortly recovered his senses, and the tumor was now somewhat diminished. He took nothing but sugared water; and, finding that perfect tranquillity alleviated the feeling of distress, and that the tumor in the neck gradually decreased, he remained perfectly quiet for two or three hours, and, as soon as he felt his usual calmness restored, he returned home. Dr. C. saw him at seven o'clock in the evening. When the patient arrived at home, he went to bed, and the shivering with which he was at first attacked gave place to abundant and general sweating, which he promoted by an additional quantity of covering. When Dr. C. visited him, his face was red and wet with perspiration; heaviness of the head; pulse large and full, beating eighty-eight. The neck was carefully examined where the tumor had formed; the part pointed out by the patient was the anterior and inferior part of the neck, inside the sterno-mastoidean muscle, and directly behind the right sterno-clavicular articulation. The tumefaction had disappeared, but still this side of the neck was fuller and softer than the other. On pressing very gently above the sternal extremity of the clavicle in the direction of the trachea, deep-seated pain was produced. Deglutition was not at all impeded, nor was any inconvenience sustained in moving the head in any direction. The patient complained only of an acute and circumscribed pain in the middle of the right breast, of an uncomfortable sensation of weight in the epigastrium, and a difficulty in breathing on the right side. He was much alarmed at his situation. When his confidence was somewhat restored by the friendly assurances of his physician, he entered into a minute detail of the manner in which he had been attacked. For some moments he spoke with vivacity, when he suddenly became pale, his voice faltered, and in a moment the skin of the neck was raised at the part before mentioned by a tumor about the size of an orange. He made repeated efforts to vomit; a cold sweat succeeded to the heat which had previously existed; the respiration was difficult and intermitting, and accompanied by severe pain upon the surface of the right breast and the lower part of the neck. He sat up in bed, leaning his body forwards, and declared that he felt himself to be dying; but, notwithstanding his extreme paleness and faintness, the pulse was not irregular, nor small, and beat the same number as before. This state of distress continued for several minutes, during which the countenance presented an alarming aspect. The tumor had much enlarged, and gave to the neck the appearance of those large goitres which occupy one half of the thyroid body; the skin covering the tumor retained its natural temperature.

The rapid development of the tumor, its external appearance, and its situation, explained the phenomena which had arisen, and shewed that it arose from a circumscribed emphysema, which was probably caused by a very limited rupture of the trachea. The extreme apprehension of the patient, much more than the real sensibility of the tumor, prevented Dr. C. from determining by pressure, whether there was the characteristic crepitation of an emphysematous tumor. The patient was enjoined to remain perfectly quiet and silent, in order that even a momentary acceleration of the respiration might be avoided. An anodyne was given, and a cold aromatic liniment, and a bandage, were applied to the tumor. Every endeavour was used to rally the drooping courage of the patient, who was dreadfully alarmed at the second attack. He passed a sleepless and agitated night; the friction was repeated early in the morning; and, when Dr. C. saw him the following day, the tumor had almost entirely disappeared, but there still remained an uncomfortable feeling of heaviness at the epigastrium, and pain in the chest, although these symptoms were also diminished, and gradually disappeared.

After a week's rest, Mr. R. was able to perform his usual business; and, during the succeeding four months, his health remained perfectly good.

Cases have been recorded of spontaneous emphysematous tumors, developed in consequence of violent efforts during respiration, but in the above instance no such cause existed. But, however the emphysema may originate, it manifests itself in the neighbourhood of the clavicles, and generally is productive of no serious event. In the above case, the particular symptoms that presented themselves led to the presumption that the air escaped in a very small quantity, and probably in consequence of a lateral rupture of the trachea, and that it had infiltrated itself into the cellular tissue which surrounds the pneumogastric nerve, from whence resulted a displacement, or elevation of the nerve, to which might be attributed the spasmodic phenomena which affected the stomach, as well as the troubled respiration. The severity of these symptoms was, in fact, in direct proportion to the volume of the tumor in the neck, so that they were aggravated as its volume increased, and diminished in intensity when the size of the swelling was diminished. Mr. C. is not aware that a similar case has been recorded; and certainly, whatever explanation may be offered of its nature, the fact is sufficiently interesting to attract attention.

---

A case recently occurred in the practice of one of the Editors, Mr. North, which somewhat resembles the above. A healthy man was sitting at table, and quietly eating his dinner of roast leg of mutton. He made no sudden movement whatever, nor was any painful or laborious effort necessary in swallowing any portion of the food of which he was partaking. He himself stated that he felt suddenly, while he was eating, a slight stiffness of the jaw on the left side, but it was too trifling to induce him to pay any attention to it, or to mention it to his family, who were sitting with him. He became somewhat alarmed, however, upon being told by his son, who was next to him at table, that his face was very much swollen, and, on putting his hand to his cheek, he felt a large tumor, about the magnitude of a middle-sized orange. He still felt no pain, nor inconvenience of any kind, excepting the mental emotion that was naturally excited by so unusual and unexpected an occurrence. Mr. North was immediately sent for: he found the tumor considerably larger than it was when first perceived, and situated exactly over the angle of the upper jaw on the left side. The temperature of the part was not increased, and its colour remained quite natural. The swelling was very slightly tender on pressure, and presented to the feel all the indications of an emphysematous tumor; its margin was, however, well defined, and there was no swelling whatever of the cheek beyond the circumference of the tumor. A cold lotion of muriate of ammonia, vinegar, and water, was applied frequently during the day, and in the course of twenty-four hours all appearance of the swelling had vanished, the patient not having been incommoded by it in any way. Some stiffness of the jaw remained for several days. Mr. North confesses he cannot explain the cause which could give rise to the sudden formation of the tumor.

As an appendix to the preceding cases, the following, which occurred in the practice of the junior Editor, may be not unworthy mention. A gentleman, while blowing his nose, suddenly found the upper eyelid enlarge; and the swelling, at first trifling, and confined to the nasal side, soon increased, and extended all over the superior palpebra, which became as large as a common hen's egg. On pressure, the tumor crepitated, and was thus shewn to be decidedly emphysematous. It subsided under the use of cold applications; but it has twice returned at distant intervals, the last time being now upwards of a year ago.

### PRACTICAL MEDICINE.

*Belladonna given to prevent Scarlet Fever.*

During an epidemic scarlatina, 120 children, from one to six years of age, took the belladonna regularly; twenty or thirty took it irregularly; from twenty-five to thirty did not take it all. Of the first number, five only were attacked with scarlatina; of the second number, eight, and of the third, eleven. Dr. H. employed a solution of two grains of extract of belladonna in an ounce of water, and he prescribed each morning as many drops as the child was years old. Of the children who died of the epidemic, not one had taken the belladonna.

For additional evidence upon this subject we refer to our Journal for January 1828, p. 67, and April 1831, p. 362.—Editors.

### PRACTICAL SURGERY.

*Danger from Wounds of large Veins.* [Extract from a Paper by M. Gensoul, describing the Cases which occurred in Paris during the Disturbances in 1831. *Gaz. Méd.*, tome i. No. 43.]

An injury of the large veins is, perhaps, a more serious accident than that of the arterial trunks. Thus, an opening of the femoral vein, particularly above the commencement of the saphena, almost always gives rise to fatal hemorrhage, if the bleeding be not arrested, or to gangrene, if the vein be obliterated. M. Roux relates a case, in his work on Operative Surgery, of a military surgeon who received, in a duel, a sword-wound, which opened the femoral vein in the groin. A ligature was applied to the injured vessel, and mortification of the limb ensued. In similar circumstances, M. R. advises immediate amputation.

A case of this kind occurred at the Hôtel Dieu of Lyons, in 1820. A soldier wounded a young man in the groin with his sabre, and he fell drenched in blood. Every endeavour was used by the persons around to restrain the hemorrhage, but in vain. The patient was taken to the hospital, and soon died. It was found, upon dissection, that the external crural vein was wounded.

### MATERIA MEDICA.

*Lunar Caustic Blister.* In the 5th vol. of the Transactions of the Medical and Physical Society of Calcutta, we find an article by J. C. Boswell, Esq. in which the lunar caustic is recommended as a substitute to cantharides for producing vesication. Mr. B. has used that remedy in pneumonia, phthisis, rheumatism, dysentery, &c. with, he says, decided utility. The blister is formed by slightly wetting the part, and then slowly drawing the stick of lunar caustic over the surface to be blistered, first longitudinally, and then across. The fluid is discharged by small punctures, in about ten hours after the application of the caustic; and no covering being used throughout, the surface becomes in two or three days sufficiently dry to admit of a second application, in the same place if necessary. Mr. B. has put on more than thirty of these blisters in the course of treating a pulmonary case, making the intervals longer as the cure proceeds.

The advantages which the nitrate of silver, applied as a blister, possesses over cantharides, according to Mr. B. appears to be that its action is more immediate, and effect more powerful; it does not affect the urinary organs, requires no dressing, is easy of application, and always available, being so easily carried about.

Mr. Twining, the intelligent secretary of the society, has appended to this paper some remarks confirmatory of the statements of Mr. Boswell. He says that he has found the caustic blisters very useful in chronic rheumatism of long duration, affecting the joints, and unattended with pyrexia, and with little or no acute local inflammation.—*American Journal of the Med. Sciences.*

---

### GEOLOGY.

### *An Account of the Ascent of the Puy de Dôme; with some Reflections upon the Geological Characters of the ancient Province of Auvergne.* By Alfred S. Taylor, Esq.

The Puy de Dôme is one of the loftiest mountains of central France, and will ever be celebrated in the records of philosophy, in consequence of its having been the spot where the variation in the pressure of the atmosphere was first demonstrated by experiment. It was simply by carrying a barometer to the summit of this mountain that Blaise Pascal first clearly established the truth of the theory of Galileo and Torricelli.*

There are, however, other circumstances which render this mountain interesting to the traveller. Its peculiarly symmetrical form, its volcanic nature, and its commanding position on an immense platform of lava, confer on it a degree of interest which other, and more lofty mountains, are very far from possessing in the eyes of the geologist.

On the 9th October, 1828, I left Clermont, in conjunction with a friend, for the purpose of examining the mineralogical characters of this mountain. Our path lay, at first, through a wild ravine, bounded by stupendous rocks of granite and gneiss. The granite, in many parts, is split into columnar masses of a somewhat regular

* This important experiment was tried in the year 1648. The barometers at the foot of the mountain, prior to the ascent, stood at 28. One of them was carried to the summit, when it fell to 24.7 inches. On descending, Pascal observed that the column of mercury proportionably increased, and it finally rose to 28. The difference of pressure at the base and summit of the mountain amounted to about 3.5 inches; and, as one inch pressure within a certain limit is equivalent to 1000 feet elevation, it was proved that the summit of the mountain was 3.500 feet above its base. The ascertained height of the Puy de Dôme is 3.565 feet.

form, and which, at a certain distance, take the appearance of castellated ruins. Large masses of granite are met with in a completely disintegrated state, crumbling in the fingers on very slight pressure. This change is ascribed by Dolomieu to the action of carbonic acid, which is constantly escaping through fissures in the volcanic soil; and he has fancifully named this species of decomposition the "*maladie du granite.*"

Crossing over several cornfields, we reached the foot of the mountain, and commenced our ascent by a somewhat broad, although a very rough and uneven, road. It was impossible to avoid remarking, as we traversed the forest which skirted its base, the curiously-coloured masses of rock which projected from the sides of the road. The first specimens which we collected were of a slate-blue colour, and bore the character of pure lithoid lava. Further on we collected others, which had a decidedly ochreous tint, varying from red to orange and yellow; but it was not until we had reached the point where the trees begin to disappear, that we met with specimens of pure cellular lava. The soil, at a certain depth, appeared to rest upon this substance, and the road was covered by the finely-powdered volcanic dust. It is difficult to understand how an individual who has traversed this spot could come to any other conclusion than that the mineral substances around have been produced by the agency of fire. They have the stamp of their volcanic origin marked as indelibly upon them as have those masses which are hourly poured forth from the craters of Etna and Vesuvius. Indeed, on mixing some of the specimens of lava from the Puy de Dôme with others subsequently collected on Vesuvius, I have found that even experienced mineralogists have been unable to determine which was the product of the modern, and which of the ancient, volcano.

We now reached an open and barren platform, where all traces of a path were lost. The weather, which had hitherto been favorable, threatened us with a storm; but still we continued our course up the side of the mountain. The prospect before us soon became singularly wild; we were placed upon a ridge of the elevation of about three thousand feet. On our right was a somewhat steep descent, and in the distance a most extensive view of the Department of Cantal, with many of the minor extinct volcanic cones bounding the horizon, and arranged in detached insulated groups. On our left, at some distance below us, was a considerable hollow, of a circular form, of great depth, and its sides clothed with the verdant grass. This is described by many, and believed by most travellers, to have been the ancient crater of the Puy de Dôme. Sheep, scattered here and there, appeared like white specks in its ample circuit, recalling forcibly to my mind the description which is left us of the crater of Vesuvius, as it appeared in the early part of the sixteenth century. Then, it is said, flocks and herds were quietly grazing amid forests and on downs, in that amphitheatre, whence torrents of volcanic matter are now periodically poured forth with such violence as to lay waste leagues of country. In the same way, the present tranquillity of this fertile province may be but the slumber of the earthquake, which ceases only to return; and a few short centuries may suffice to bring about a renewal of the work of devastation, to scatter the habitations of man, and to teach him that he is not secure from these mysterious operations of nature, although living surrounded by the fruits of industry and civilization!

The further ascent of the mountain we found to be somewhat difficult, on account of the great looseness of the soil, which seemed to be formed entirely of a black pulverulent mould, probably arising from the disintegration of lava. In the immediate neighbourhood of the spot where we had rested, we found projecting from the side of the mountain a ridge of granite, which, however, was so friable that it readily crumbled to dust on pressure. The existence of granite at the height of three thousand feet on this mountain, is a fact which I do not remember to have seen noticed by any traveller in this quarter. It appeared as if it had been formerly covered by lava, but which, in the course of time, had become decomposed and washed away.

The cold now became severe, the thermometer sinking ten degrees below the freezing-point. The clouds were carried past us with the rapidity of lightning, completely obscuring our progress; and, in the meantime, we were obliged to cling to the projecting masses of lava and granite, to prevent ourselves from being hurled by the wind to the base of the mountain. We at length, after considerable difficulty, gained the summit; but, in consequence of the extreme violence of the wind, we found it impossible to remain there: we therefore sheltered ourselves by retiring immediately below its level, and lying down on the declivity. This part of the mountain, as indeed the whole, so far as we could observe, is covered with a light thin grass, loosely adherent to the mould. There are no parts bare or exposed, except those where the granite projects beyond the symmetrical outline. The soil, when violently struck, gave a peculiar hollow sound, a circumstance which has led some to suppose that its summit forms merely a crust or coating to a hidden abyss. This, however, is a phenomenon which appears to depend upon the loose and disintegrated state of the soil.

The weather now began to clear up, and, for the first time, we obtained a glimpse of the seemingly boundless landscape before us. The scene which presented itself was wild and beautiful in the extreme. The vast mass of the Puy de Dôme lay at our feet, in an almost perpendicular wall; the supposed crater had dwindled to a mere hollow; and the forests, through which we had passed in commencing the ascent, now appeared like indistinct green-coloured patches on the dark brow of the mountain. From our shelter, as from a central point, we looked down upon the whole of the province of Auvergne. Before us lay the city of Clermont, scarcely to be distinguished, on account of its diminutive appearance. On the one hand, the windings of the Allier, then forming but a bright streak in the landscape, and taking its course northward to join the Loire; on the other, a series of volcanic cones, stretching into the department of Cantal, and passing onwards to unite with the mountains of Languedoc, among which were particularly to be distinguished the Mont D'Or and the Plomb de Cantal. It would indeed be impossible to convey in language an idea of the beauty or splendour of the scene which unfolds itself to the view from the summit of this mountain; but, to appreciate it, it is necessary that a man should travel to study nature, and not to gratify an idle or unmeaning curiosity.

The descent of the mountain, owing to our having taken an unfrequented route, we found much more difficult than the ascent; and more than once we were nearly precipitated to the base, by the deluge of loose stones and earth which are detached at each step.

Some have doubted whether Auvergne were a volcanic district or not; whether the so-called beds of lava observed in it were produced by the action of fire, or whether they resulted from a deposition by water. The indefatigable Dolomieu appears to have been the first to set the philosophical world right upon this subject; and his memorable answer to a disciple of the Wernerian school soon led to the most important investigations.* Those who visited the spot, with a few exceptions, came back satisfied that no other reasonable or satisfactory explanation of the nature of the locality could be offered.

The conoid forms of the mountains; the physical and chemical properties of the great masses of mineral matter of which they are constituted; and, lastly, the presence of sulphur, and of other substances of known volcanic nature, establish the correctness of the theory respecting their igneous origin. But one of the most striking and extraordinary proofs which can be offered, is to be seen in the Museum of the Jardin des Plantes at Paris.†

Admitting then the truth of this position, we cannot fail to be astonished at the stupendous power which, in the primitive age of the world, must have been exerted by the volcanic mountains of this part of France. The whole of the province of Auvergne may be truly said to have been raised out of the bowels of the earth, and it has, no doubt, purchased its present fertility at the expense of widely-spreading desolation. Recent researches have shewn that there are no less than one hundred and fifty extinct craters scattered over its surface, of which seventy are to be met with within the space of a league and a half. These craters are now filled up,

* It is said that Dolomieu was, on one occasion, arguing with an advocate of the Neptunian hypothesis, respecting the origin of the lava of Auvergne. His opponent, who had studied geology in the closet only, and not in nature, received a reply ("allez voir") to all his ingenious arguments, which at once silenced him.

† This specimen consists of a group of sticks united together by flakes of lava. The vegetable matter appears to have been perfectly carbonised, and to have become incrusted by the lava while semifluid. It was found, not many years since, in the immediate neighbourhood of the Puy de Dôme.

and have been for the most part converted into vineyards, or pastures; and many a cottager has built his hut on the verge of a volcanic mouth, unconscious of the foundation prepared for him by the hand of nature!

Of all these volcanic cones, the Puy de Dôme is the most worthy of observation, being insulated upon a vast platform of lava, and forming a striking contrast with the minor cones in the neighbourhood. The knowledge of its present state, combined with the recollection of what it has been, a gigantic engine of volcanic power, mighty enough to produce its own stupendous mass, and to cover upwards of a hundred square leagues of the surrounding country with lava, will lead the reflecting mind to the conclusion that the resources of nature are indeed vast beyond the comprehension of man. Next to the Puy de Dôme comes the Mont D'Or, so called from the yellow colour of its lava; and, lastly, the Plomb de Cantal, which is situated in an adjoining department.

It may seem extraordinary, with such clear evidence before us of past volcanic agency, that we possess no record or tradition which can lead to a conjecture respecting the time at which these craters were in a state of eruption. It has been vaguely stated, that they were still in activity about the ninth century of the Christian era; but, if this had really been the case, it is not probable that the fathers of the church would have passed the phenomena over in silence, ready as they were, in that age of superstition and tyranny, to make use of every extraordinary natural occurrence for some specific religious or political purpose. Certainly, any such event would have found its place in history; but, by history or tradition, the statement is altogether unsupported; and hence it may be safely rejected. If a speculation might be hazarded from the appearance of the lower strata of lava, I should refer the epoch of their eruptions to a period even more remote than the Noachian deluge. Then, probably, only the centre of that which we now denominate France, rose above the waters of the ocean, as a small insulated volcanic spot, like some of those isles now met with in the Pacific and Indian Oceans. At this time, probably, the whole of Europe was below the level of the sea, the primeval summits of the Alps alone rising above its waters. Such a speculation may seem fanciful in the extreme; but there are geological data which would lead to conclusions perhaps even more wild and untenable than this. It would be idle to inquire why these volcanic mountains should have ceased their eruptions. They have performed nature's work; they have fertilized the earth, and, for centuries, have served as a nidus of vegetation; while, on the other hand, it is probable that the active volcanoes with which we are acquainted now take that share in the processes of destruction and vivification which these have taken formerly, and of which they have left such imperishable evidence!

But it may be asked, is it probable that these volcanoes will ever resume their destructive agency? Such a question can only be answered analogically; and when we reflect that Vesuvius was not believed to be a volcano up to the period of its eruption, in the reign of Titus; that there was no record of its ever having been in a state of eruption prior to that epoch, although we know, from an examination of the surrounding country, and from an inspection of the materials employed in the buildings of Pompeii, that the eruption by which that city was laid desolate was only one of a series, of which the chain was for a time broken. Clermont is now what Pompeii was in the time of the unsuspecting Romans, who little imagined that they had founded a city on the very brink of a volcano, and of materials which afforded the most irrefragable evidence of the real nature of the locality. The Auvergnois are now enjoying that security which ignorance so often entails; they have no knowledge of the nature of the district which they inhabit; and to affirm that the Puy de Dôme had formerly been a volcanic mountain, or that they owed the actual fertility of their vineyards to the disintegrated lava-beds which have poured down its sides, would be sufficient, in their eyes, to stamp the individual for a madman. Such is the state of the human mind: the most sublime truths will be for ages regarded by the majority of men as visionary subtleties, and they will only be aroused from this dream of ignorance by the practical application which may be made of them to the common affairs of life, or by the direct evidence of impending danger!

An examination of the province of Auvergne has settled the question which was formerly much disputed, relative to the source of the lava which issues from the crater of a volcano. Most of those which are at present in activity throughout the globe, and indeed the extinct craters of the Rhine, of Hungary, of Palestine, and Italy, are chiefly situated in secondary or tertiary districts, or dispersed in insulated patches on the surface of the ocean. But in central France we find the craters are placed upon a vast bed of granite, which, as is well known, is the lowest of all the strata of the earth to which the excavations of man have hitherto extended. In this fact, then, we have direct evidence that lava must issue from depths considerably below the crust of the earth; that, in short, it must proceed from a source to which it is impossible to trace it; and therefore, with respect to the manner of its production, we must yet be content to remain in ignorance!

---

### CHEMISTRY.

*Alteration of Sound in Vessels containing Carbonic Acid Gas.* By Alfred S. Taylor, Esq., Lecturer on Chemistry in Guy's Hospital.

It was first remarked by Chladni, that when a liquid, in a state of effervescence, was poured into a glass vessel, the glass no longer gave its peculiar ringing sound when struck by a hard body, as by a piece of metal. The sound became dull and dead, but reacquired its peculiar sharpness in proportion as the effervescence ceased. This experiment may be familiarly performed on a glass of soda-water, or champagne; and it will be immediately observed, that the alteration in the sound is great in proportion to the violence of the effervescence. If the effervescence has ceased, upon restoring this by dissolving in the liquid a quantity of powdered sugar, the phenomenon will be again remarked as strikingly as before.

The explanation afforded by Dr. Brewster, in his interesting work on Natural Magic, appears perfectly satisfactory. "Sound," he observes, "moves with different velocities, through media of different densities; hence the wave which produces the sound will be partly reflected in passing from one medium to another, and the direction of the transmitted wave changed. Thus, in passing through such media, different portions of the wave will reach the ear at different times, and thereby destroy the sharpness and distinctness of the sound." In the instance above cited, we have the sound transmitted partly by the stratum of carbonic acid covering the surface of the liquid, and partly by the surrounding air; and, on account of the difference of density in the two media, we have so great an interruption to the uniformity and regularity of the vibrations, that the sound becomes changed in the manner already described.

During the last winter, I had occasion to apply the knowledge of this principle to a use which it may not be uninteresting to notice. Some jars, containing carbonic acid gas, prepared for a lecture, had by accident become intermixed with other jars standing on the table, and containing other gases. Some doubts arose as to which of these vessels contained carbonic acid; but the question was speedily determined by gently tapping the exterior of the jars with an iron nail, the sound emitted by the vessels containing the carbonic acid forming a most striking contrast with that given out by the others. The knowledge of this fact may not be without its use to those who are engaged in experiments on the gases.

---

### *On the Preparation of Carbonic Oxide Gas.*

By A. S. Taylor, Esq.

This gas appears to have been first obtained by its discoverer, Dr. Priestley, in the distillation of charcoal with oxide of zinc; and the process for its preparation seems to have undergone but very little alteration since his time. In our most popular works on Chemistry, at least in those of Henry and Brande, the directions for its preparation are extremely complex, and are unfitted for its extemporaneous production; as, for example, during a lecture. A strongly-heated furnace, an earthen or iron retort, &c., with a gasometer, are, according to the processes given by these chemists, indispensably necessary to the experimentalist; whereas, in the process given below, the gas may be readily produced in a few seconds, and on the table of the laboratory or lecture-room.

A small retort is to be filled to about one third with crystals of oxalic acid. A quantity of strong sulphuric acid is then to be added, but not more than is sufficient to moisten the crystals. The heat of a spirit-lamp may now be applied, and the gas will come over abundantly, and may be collected in the ordinary way over a water-bath.

The decomposition which here takes place will be readily understood. Oxalic acid consists of carbon and oxygen, in the proportions intermediate between those of carbonic acid and carbonic oxide. The crystals contain about one half of their weight of water, and the sulphuric acid acts by taking this water, and liberating the carbon and oxygen, under the form of the two compounds mentioned.

The gas obtained in this way is, therefore, always combined with carbonic acid; but the same fact is invariably observed with regard to the gas obtained by distilling an earthy carbonate with iron filings. To procure the carbonic oxide perfectly pure, it will be necessary, in either case, to allow it to pass over a cream of lime, or through a solution of caustic potash.

In a work recently published by Dumas, I find the binoxalate of potash recommended; the salt being treated with sulphuric acid, and heated in a retort. I am not aware that this possesses any advantages over the method which is above described, and which I have been for a long time in the habit of employing; but either the one or the other must be regarded as much more commodious, and less troublesome to the experimentalist, than the processes ordinarily recommended in works upon the science.

